# Pretreatment of Cottage Cheese to Enhance Biogas Production

**DOI:** 10.1155/2014/374562

**Published:** 2014-06-03

**Authors:** Vidhya Prabhudessai, Bhakti Salgaonkar, Judith Braganca, Srikanth Mutnuri

**Affiliations:** Applied and Environmental Biotechnology Laboratory, Department of Biological Sciences, Birla Institute of Technology and Science, Pilani, K. K. Birla Goa Campus, Goa 403726, India

## Abstract

This study evaluated the possibility of pretreating selected solid fraction of an anaerobic digester treating food waste to lower the hydraulic retention time and increase the methane production. The study investigated the effect of different pretreatments (thermal, chemical, thermochemical and enzymatic) for enhanced methane production from cottage cheese. The most effective pretreatments were thermal and enzymatic. Highest solubilisation of COD was observed in thermal pretreatment, followed by thermochemical. In single enzyme systems, lipase at low concentration gave significantly higher methane yield than for the experiments without enzyme additions. The highest lipase dosages decreased methane yield from cottage cheese. However, in case of protease enzyme an increase in concentration of the enzyme showed higher methane yield. In the case of mixed enzyme systems, pretreatment at 1 : 2 ratio of lipase : protease showed higher methane production in comparison with 1 : 1 and 2 : 1 ratios. Methane production potentials for different pretreatments were as follows: thermal 357 mL/g VS, chemical 293 mL/g VS, and thermochemical 441 mL/g VS. The average methane yield from single enzyme systems was 335 mL/g VS for lipase and 328 mL/g VS for protease. Methane potentials for mixed enzyme ratios were 330, 360, and 339 mL/g VS for 1 : 1, 1 : 2, and 2 : 1 lipase : protease, respectively.

## 1. Introduction

Food waste is the single largest component of waste stream by weight. About 135.5 million tons per year of municipal solid waste is generated in India and food waste alone constitutes about 30–40% [1]. Anaerobic digestion is a proven technology that offers significant environmental benefits and has been considered as one of the most viable options for managing solid organic waste [2]. Anaerobic digestion is a process, where complex particulate organic material is broken down into simpler soluble compounds which are taken up by microbial cells and ultimately converted into methane and carbon dioxide.

Food waste is characterized by its high organic content, most of it being composed of easily biodegradable compounds carbohydrates, proteins, and, in some cases, small amount of lipids. The anaerobic biodegradability of organic matter depends on its composition and the amount of methane produced depends on the biochemical nature of the waste [3]. For instance, carbohydrates, proteins, and fats show different methane production rates [4]. Food waste contains variable type and amount of organic matter whose behaviour in digester depends on the biodegradation of organic pools characterized by different methane production rates. Although food waste has been regarded as readily biodegradable because of its high volatile fraction (90% of total solids), its hydrolysis reaction is still a rate limiting step [5]. Enhancement of the hydrolytic reaction during anaerobic digestion could shorten the hydraulic retention time and thus improve the economics of the process. During recent years, various studies have been conducted on pretreatment of food waste, such as mechanical and sonication [6], thermal [7, 8], acid [9], alkaline [10], and enzymatic [5, 11, 12].

We had set up a horizontal plug flow type of anaerobic digester handling one ton of food waste/day and generating 60 m^3^ of biogas/day. The food waste for this plant is from the institute's cafeteria catering to 2500 students. The commonly seen undigested solid fractions in the outlet of the digesters are cottage cheese, whole potatoes, and whole eggs. Studies have shown that digestate can still contain a high biogas potential, mainly as a consequence of residual and undigested volatile solids [13]. Digested solid fraction, with its biogas and methane potential, could be used as a substrate for anaerobic digestion [14]. Utilizing digested, separated solid fraction in this manner would capture residual methane and, consequently, could reduce GHG emissions [15]. An attempt has been made in this work by conducting lab scale studies to improve anaerobic digestion by pretreating cottage cheese using thermal, chemical, thermochemical, or enzymatic methods.

These pretreatments could improve the waste stabilization and methane production but their application should be proved to be commercially viable in relation to the additional processing costs [16]. Due to high fat and protein content, cottage cheese can be considered as a good substrate for anaerobic digestion process. For enzymatic treatment, we have used enzymes from extreme halophiles as extremozymes are more stable in harsh conditions.

## 2. Materials and Methods

### 2.1. Screening of Enzyme Producing Haloarchaea

The first stage in this study was screening of extreme halophiles isolated from solar saltern for production of protease and lipase. The cultures were grown on NTYE (NaCl, tryptone, and yeast extract) medium. The culture medium for enzyme production was composed of (w/v) NaCl 200 g/L, MgCl_2_·6H_2_O 13 g/L, KCl 4 g/L, CaCl_2_·H_2_O 1 g/L, NaHCO_3_ 0.2 g/L, NH_4_Cl_2_ g/L, FeCl_3_·6H_2_O, KH_2_PO_4_ 0.5 g/L, pH 7.0 inoculum density 2% (v/v). For production of protease skim milk was used as substrate and for lipase olive oil was used as substrate.

### 2.2. Screening Method Zone of Clearance

The organisms were allowed to grow on agar plates containing substrate for particular enzymes, that is, skim milk for protease and olive oil for lipase, and incubated at room temperature. The enzymes used in this study and their reported activities were protease, 125 units/mL, and lipase, 150 units/mL. The activity of protease was checked with skim milk as substrate and activity of lipase was checked with olive oil as substrate. For measuring protease and lipase activity the raw enzyme used for the assay was isolated from the culture broth following separation of cells. The culture medium was centrifuged at 8,000 rpm for 20 minutes at 4°C and the cell-free supernatant was used as the source of enzyme.

### 2.3. Concentration of the CFS

The cell-free supernatant (CFS) of the isolates obtained as described above was subjected to ultrafiltration. Briefly, 50 mL of the CFS obtained was concentrated 10 times (i.e., 5 mL) of its original volume using nominal molecular weight cut-off (NMWCO) 3.0 kDa cellulose membrane in a stirred ultrafiltration cell (Model 8050, Millipore, USA).

### 2.4. Substrate

Cottage cheese separated from the undigested solid fraction food waste was used as the substrate. The cottage cheese was collected from the outlet of the plug flow digester treating food waste.

### 2.5. Pretreatments

 Thermal: the substrate was autoclaved at 15 psi and 120°C for 20 minutes.
 Chemical: the substrate was treated with sodium hydroxide 0.5 M.
 Thermochemical: the substrate was first autoclaved at 15 psi and 120°C for 20 minutes and then 0.5 M sodium hydroxide was added.
 Enzymatic: first, the single enzyme hydrolysis of cottage cheese using protease and lipase was carried out at different concentrations from 0.02 to 0.5% (v/v) with each enzyme. Secondly, three levels of mixed enzyme ratio were tested with 0.04% of mixed enzyme ratio: 1 : 1, 1 : 2, and 2 : 1 of protease and lipase, respectively, to determine the optimal enzyme mixture ratio.

### 2.6. Biochemical Methane Potential Assays of Pretreated Cottage Cheese

Methane potential was determined in batch assays as described in [17–19]. The inoculum for batch assays was the effluent from a mesophilic anaerobic reactor treating food waste. The reactors were supplemented with nutrients, trace elements, and bicarbonate. Finally, the reactors were made up to the working volume of 0.1 L with distilled water and the headspace was flushed with nitrogen. A control without substrate was also set up to account for the endogenous biogas produced from the inoculum. All the experiments were carried out in duplicates. The bottles were shaken manually once a day. Biogas production was measured using water displacement technique. Gas samples were taken periodically for composition analysis by gas chromatography using hydrogen as carrier gas. The calculated biogas production is also corrected for blank biogas production.

### 2.7. Analysis

Total solids and volatile solids were measured in accordance with standard methods [20]. The Chemical Oxygen Demand measurement is performed on fresh waste. Prior to use the substrates were ground in a blender to give a fraction with particle size less than 2 mm. The substrate (1 g) is then suspended in 1 L mL of distilled water and stirred on a magnetic stirrer for one hour, and the COD of the suspension is measured as described in [21]. Gas samples were taken periodically for composition analysis. The samples were analysed with a gas chromatograph (GC-7610, Chemito) equipped with thermal conductivity detector. The carrier gas was hydrogen. The oven, injector, and detector temperatures were 80, 150, and 250°C, respectively.

## 3. Results and Discussion

### 3.1. Screening of Protease and Lipase

Four strains were selected and screened for production of lipase and protease enzymes. It was found that BK-11 and BK-20 were shown to produce larger zone of clearance (Table 1). These two strains were selected for further work.

### 3.2. Characteristics of the Substrate

The main characteristics of cottage cheese used in the experiment are reported in Table 2.

### 3.3. Effect of Pretreatment on Solubilisation of Cottage Cheese

Four different pretreatments (thermal, chemical, thermochemical, and enzymatic) were used in order to hydrolyse cottage cheese. The soluble COD of cottage cheese increased with each pretreatment compared to the untreated sample. The most effective pretreatments were thermal and enzymatic. Chemical and thermochemical pretreatments were less effective in terms of solubilisation. Cottage cheese consists of 25–27% of fats. Sodium hydroxide has been reported more efficient in hydrolysing proteins and carbohydrates than lipids [22] which could explain lesser solubilisation of cottage cheese with alkali treatment. Biological and physiochemical pretreatments promote the substrate hydrolysis, breaking down the polymer chain into soluble components [23]. For enzymatic pretreatments, an increase in soluble COD was observed with increasing concentrations of enzymes. Pretreatment with protease showed higher solubilisation percentage in comparison with lipase (Table 3).

### 3.4. Biogas Production and Methane Yield during Pretreatments

**Figure 1:**
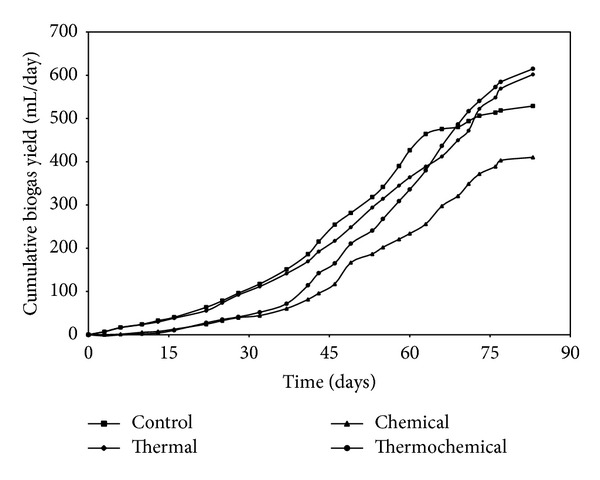
Cumulated biogas yield at different pretreatments.

**Figure 2:**
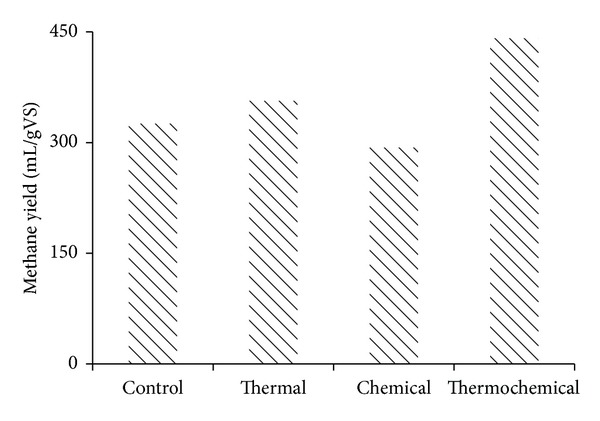
Cumulated methane yield at different pretreatments.

For the different pretreatments studied, biogas production started immediately with thermal pretreatment, but thermochemical and chemical pretreatments showed a lag phase of around 15 days. The highest cumulative biogas production was obtained in thermochemical pretreatment, 615 mL/g VS, followed by thermal treatment 602 mL/g VS (Figure 1). For the lowest production, 410 mL/g VS was observed in chemical treatment. Thermal pretreatment is capable of speeding the hydrolytic phase of anaerobic digestion favouring organic molecule degradation and accelerating the bacterial metabolic process [24]. Alkaline pretreatment generally requires longer reaction times compared to other pretreatment methods [25]. The highest methane yield was obtained with thermochemical pretreatment 441 mL/g VS followed by thermal alone (357 mL/g VS) whereas lower yield was obtained in chemical pretreatment 293 mL/g VS added (Figure 2). Although thermochemical pretreatment of cottage cheese showed high methane potential (441 mL/g VS added), the methane production started after a lag phase of 2–15 days most likely due to inhibitory compounds of alkali treatment. A disadvantage of alkaline pretreatment is the generation of irrecoverable salts and/or the incorporation of salts into the substrate during pretreatment reactions [26].

### 3.5. Single Enzyme Treatment (Lipase)

**Figure 3:**
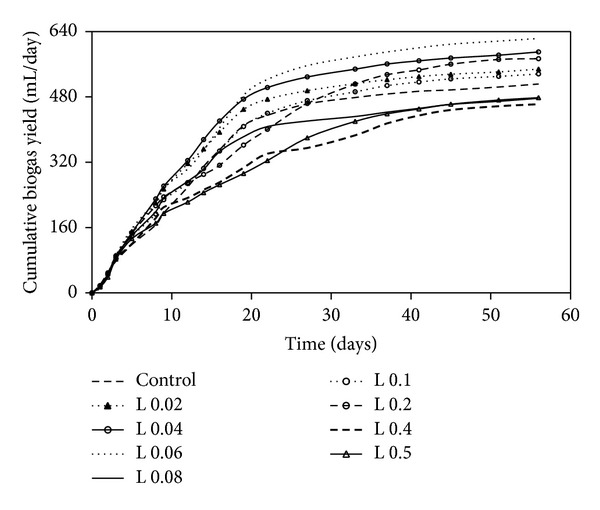
Cumulated biogas yield at different concentrations of lipase.

In single enzyme treatment with lipase, biogas production followed same pattern across all samples except the samples with 0.02%, 0.04% and 0.06% lipase wherein the maximum biogas production was faster (Figure 3). With the lowest enzyme addition there was a significantly higher yield than for the experiments without enzyme additions. The highest enzyme dosages decreased methane yield from cottage cheese. The increase in solubilized COD from pretreatment may not be inhibitory but can increase the organic loading to the methanogens and overload the anaerobic digester [27]. The maximum biogas production of 623 mL/g VS was observed at 0.06%. The addition of enzymes gave a slight increase in the initial methane production rate for different concentrations of enzymes compared to the control (Table 4). The average methane production was 335 mL/g VS. The addition of enzymes in anaerobic digesters treating food processing waste resulted in improved digestion and biogas production [28]. Cottage cheese is a product of dairy industries, and it has been reported that lipases are very promising alternative for degrading lipid rich wastewater generated by dairy and slaughterhouse industries [29].

### 3.6. Single Enzyme Treatment (Protease)

**Figure 4:**
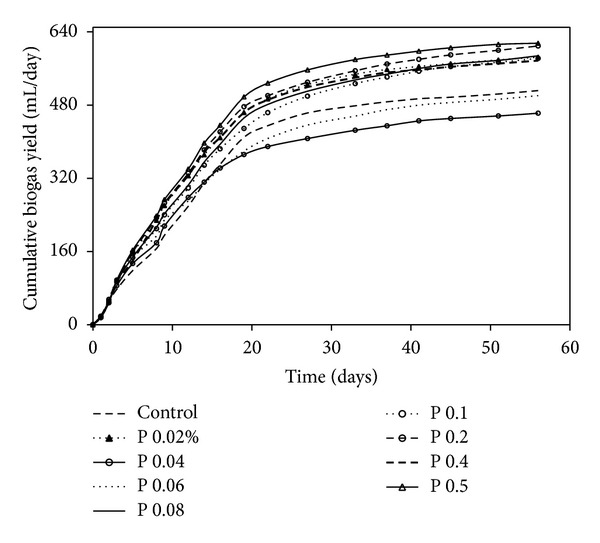
Cumulated biogas yield at different concentrations of protease.

In single enzyme treatment with protease, biogas production followed same pattern across all samples except the samples with 0.08% and 0.5% protease wherein the maximum biogas production was faster (Figure 4). In case of protease enzyme, an increase in concentration of the enzyme gave higher biogas yield. However, the average methane production was 328 mL/g VS which is slightly lower than that obtained in single enzyme treatment with lipase (Table 4).

### 3.7. Mixed Enzyme Pretreatment

**Figure 5:**
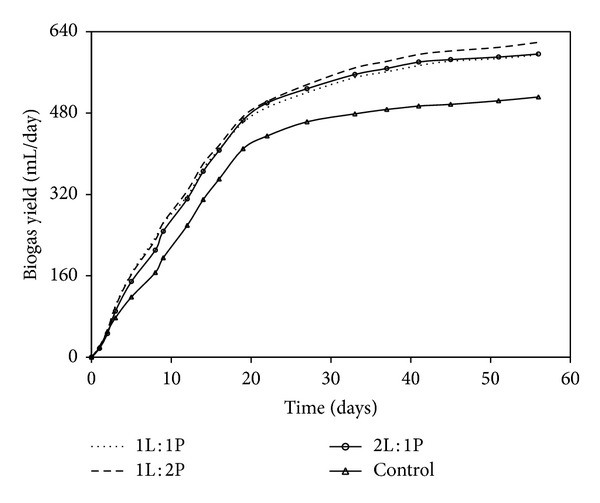
Cumulated biogas yield at different mixed enzyme ratio.

For mixed enzyme pretreatment two enzyme combinations with three different ratios 1 : 1, 1 : 2, and 2 : 1 (lipase : protease) were investigated using an equivalent dosage of 0.04% (v/v) to evaluate the effect of mixed enzyme ratio on methane production. 526, 571, and 539 mL/g VS CH4 were observed at 1 : 1, 1 : 2, and 2 : 1 lipase : protease, respectively. For all three mixed enzyme ratios, methane production was higher than those of single enzyme treatments. Although different ratios of enzyme additions did not show much significance, the biogas production rate and biogas yield were higher compared with the control (Figure 5). As expected from single enzyme pretreatment results 1 : 2 ratio of lipase : protease showed higher methane production in comparison with 1 : 1 and 2 : 1 ratios.

**Figure 6:**
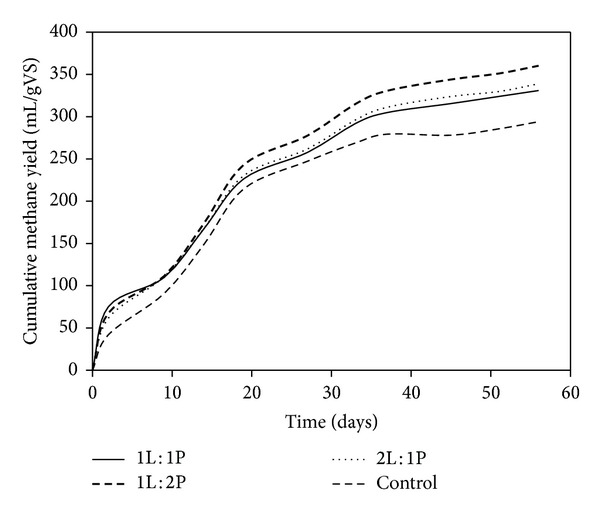
Cumulated methane yield at different mixed enzyme ratio.

In single enzyme pretreatments, we observed that the average methane production was similar, that is, 335 mL and 328 mL for lipase and protease, respectively (Figure 6). However, in the case of mixed enzyme system, pretreatment at 1 : 2 ratio of lipase : protease showed higher methane production than the 1 : 1 and 2 : 1 ratios. As discussed earlier, increase in soluble COD can increase the organic loading to the methanogens and overload the anaerobic process. At higher concentrations of enzymes inhibitions have been reported for meat processing waste [29].

## 4. Conclusion

The pretreatments studied (thermal, chemical, thermochemical, and enzymatic) effectively hydrolysed cottage cheese into soluble organic compounds. Enzymatic and thermochemical pretreatments were the most effective pretreatments for cottage cheese. Chemical pretreatment showed the poorest performance in terms of both solubilisation and biogas production. High temperature required for thermochemical pretreatment would likely raise the economic and energy dynamics of the process. Moreover, the enzymes were especially suitable for protein and lipid rich cottage cheese with low dose requirement. Cell-free enzymes offer several advantages in the treatment of waste especially to reuse the separated solid fraction as a feedstock for methane production.

## Figures and Tables

**Table 1:** Archeal strains producing protease and lipase.

Archeal strain	Protease	Lipase
BK6	−	−
BK7	−	−
BBK1	+	−
BK11	+	+ +
BK18	+	+
BK19	+	+
BK20	+ +	+

**Table 2:** Characteristics of the substrate.

Parameters		Pretreatments	SCOD (mg/L)	Biogas (mL/g VS)	Methane (mL/g VS)
TS	46.74%	Thermal	2640	601 ± 8.1	357 ± 1
VS	46.09%	Chemical	1200	410 ± 24.7	293 ± 0.8
VS/TS	0.96%	Thermochemical	1360	614 ± 37	441 ± 1.3
SCOD	860 mg/L				
Fats	25–27%				
Proteins	17-18%				
Moisture	53.26%				
pH	5.5				

**Table 3:** Solubilisation during enzymatic pretreatment.

Concentration of enzymes (%)	Lipase	Protease
0.02	1360	480
0.04	1840	2080
0.06	2320	2160
0.08	2560	2080
0.1	2640	1760
0.2	2800	3280
0.4	3440	3680
0.5	4240	4000

**Table 4:** Cumulated methane yield at different concentrations of lipase and protease.

Concentration of enzymes (%)	Lipase	Protease
0.02	327	307
0.04	312	337
0.06	311	396
0.08	350	329
0.1	339	344
0.2	348	318
0.4	318	320
0.5	320	328

## References

[1] Kashyap DR, Dadhich KS, Sharma SK (2003). Biomethanation under psychrophilic conditions: a review. *Bioresource Technology*.

[2] Khalid A, Arshad M, Anjum M, Mahmood T, Dawson L (2011). The anaerobic digestion of solid organic waste. *Waste Management*.

[3] Buffiere P, Loisel D, Bernet N, Delgenes J-P (2006). Towards new indicators for the prediction of solid waste anaerobic digestion properties. *Water Science and Technology*.

[4] Angelidaki I, Sanders W (2004). Assessment of the anaerobic biodegradability of macropollutants. *Reviews in Environmental Science and Biotechnology*.

[5] Kim HJ, Kim SH, Choi YG, Kim GD, Chung H (2006). Effect of enzymatic pretreatment on acid fermentation of food waste. *Journal of Chemical Technology and Biotechnology*.

[6] Hidalgo D, Sastre E, GÓmez M, Nieto P (2012). 8Th iwa symposium on waste management problems in agro-industries- agro’2011: evaluation of pre-treatment processes for increasing biodegradability of agro-food wastes. *Environmental Technology*.

[7] Wang J-Y, Liu X-Y, Kao JCM, Stabnikova O (2006). Digestion of pre-treated food waste in a hybrid anaerobic solid-liquid (HASL) system. *Journal of Chemical Technology and Biotechnology*.

[8] Ma J, Duong TH, Smits M, Verstraete W, Carballa M (2011). Enhanced biomethanation of kitchen waste by different pre-treatments. *Bioresource Technology*.

[9] Karlsson A, Elertsson J (2012). Addition of HCl as a means to improve biogas production from protein-rich food industry waste. *Biochemical Engineering Journal*.

[10] Eom CY, Lim J, Kim JY, Cho HS Effect of alkaline pre-treatment of food waste on solubilization and biodegradability for anaerobic digestion.

[11] Rintala JA, Ahring BK (1994). Thermophilic anaerobic digestion of source-sorted household solid waste: the effects of enzyme additions. *Applied Microbiology and Biotechnology*.

[12] Moon HC, Song IS (2011). Enzymatic hydrolysis of foodwaste and methane production using UASB bioreactor. *International Journal of Green Energy*.

[13] Hansen TL, Sommer SG, Gabriel S, Christensen TH (2006). Methane production during storage of anaerobically digested municipal organic waste. *Journal of Environmental Quality*.

[14] Menardo S, Balsari P, Dinuccio E, Gioelli F (2011). Thermal pre-treatment of solid fraction from mechanically-separated raw and digested slurry to increase methane yield. *Bioresource Technology*.

[15] Amon B, Kryvoruchko V, Amon T, Zechmeister-Boltenstern S (2006). Methane, nitrous oxide and ammonia emissions during storage and after application of dairy cattle slurry and influence of slurry treatment. *Agriculture, Ecosystems and Environment*.

[16] Esposito G, Frunzo L, Giordano A, Liotta F, Panico A, Pirozzi F (2012). Anaerobic co-digestion of organic wastes. *Reviews in Environmental Science and Biotechnology*.

[17] Owens JM, Chynoweth DP (1993). Biochemical methane potential of municipal solid waste (MSW) components. *Water Science and Technology*.

[18] Hansen TL, Schmidt JE, Angelidaki I (2004). Method for determination of methane potentials of solid organic waste. *Waste Management*.

[19] Angelidaki I, Alves M, Bolzonella D (2009). Defining the biomethane potential (BMP) of solid organic wastes and energy crops: a proposed protocol for batch assays. *Water Science and Technology*.

[20] APHA (1998). *Standard Methods for the Examination of Water and Waste Water*.

[21] Raposo F, de la Rubia MA, Borja R, Alaiz M (2008). Assessment of a modified and optimised method for determining chemical oxygen demand of solid substrates and solutions with high suspended solid content. *Talanta*.

[22] Luste S, Luostarinen S, Sillanpää M (2009). Effect of pre-treatments on hydrolysis and methane production potentials of by-products from meat-processing industry. *Journal of Hazardous Materials*.

[23] Vavilin VA, Fernandez B, Palatsi J, Flotats X (2008). Hydrolysis kinetics in anaerobic degradation of particulate organic material: an overview. *Waste Management*.

[24] Kaparaju PLN, Rintala JA (2005). The effects of post-treatments and temperature on recovering the methane potential of >2 mm solid fraction of digested cow manure. *Environmental Technology*.

[25] Chandra R, Takeuchi H, Hasegawa T, Kumar R (2012). Improving biodegradability and biogas production of wheat straw substrates using sodium hydroxide and hydrothermal pretreatments. *Energy*.

[26] Yi Z, Zhongli P, Ruihong Z (2009). Overview of biomass pre-treatment for cellulosic ethanol production. *International Journal of Agricultural and Biological Engineering*.

[27] Carlsson M, Lagerkvist A, Morgan-Sagastume F (2012). The effects of substrate pre-treatment on anaerobic digestion systems: a review. *Waste Management*.

[28] Parawira W (2012). Enzyme research and applications in biotechnological intensification of biogas production. *Critical Reviews in Biotechnology*.

[29] Mendes AA, Castro HF, Pereira EB, Castro HF (2006). Effect of the enzymatic hydrolysis pretreatment of lipids-rich wastewater on the anaerobic biodigestion. *Biochemical Engineering Journal*.

